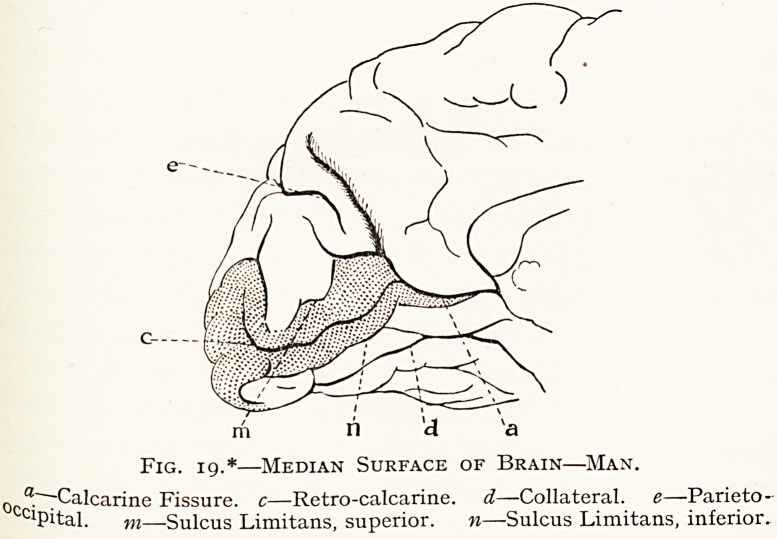# The Long Fox Lecture: The Evolution of the Sense of Sight

**Published:** 1915-12

**Authors:** F. Richardson Cross

**Affiliations:** Senior Surgeon to the Bristol Eye Hospital; Consulting Surgeon to the Bristol Royal Infirmary


### THE LONG FOX LECTURE:

THE TWELFTH ANNUAL LECTURE ARRANGED BY THE COMMITTEE OF THE LONG FOX LECTURE,

DELIVERED IN THE PHYSIOLOGICAL LECTURE THEATRE OF THE UNIVERSITY OF BRISTOL ON DECEMBER 1ST, 1915.

THE VICE-CHANCELLOR *in the Chair*.

BY

F. RICHARDSON CROSS, M.B. Lond., F.R.C.S.,

*Senior Surgeon to the Bristol Eye Hospital;*
*Consulting Surgeon to the Bristol Royal Infirmary.*

ON

### THE EVOLUTION OF THE SENSE OF SIGHT.

---

EDWARD LONG FOX was born in 1832; he died on March 28th, 1902. Having obtained a First Class in the Natural Science Schools at Oxford, he studied medicine in Edinburgh and London. He graduated M.B. in 1857, and the same year was appointed Physician of the Bristol Royal Infirmary, which post he retained until 1877. His grandfather, Edward Long Fox, had held the same position for thirty years.

He was President of the British Medical Association in 1894.

He was a student of science and a sound practitioner of medicine, ever extremely attentive to his cases whether in hospital or in private, and he was a personal friend as well as adviser to his patients. He published many valuable papers on medical subjects, and would have done even more had it not been for gout and indifferent health. He was for several years the acknowledged head of the medical profession in Bristol, and possessed the full confidence of all his colleagues. Generous in his hospitality, and in his charitable support of hospitals and other deserving objects, his influence and effort were always available for any good work, and he helped in secret many poor and struggling individuals. He was always kind and helpful to young men starting in the profession, and I am indebted to him for very many acts of kindness and hospitality.

Dr. Fox was an exceptionally handsome man with a great charm of manner, and at his death a very large circle lost a beloved personal friend, the profession of medicine one of its most trusted leaders, and the City of Bristol an honoured and valuable fellow-citizen.

---

In the lowest forms of animal life the whole surface is equally influenced by light. The most simple organ of vision would arise in some limited area often with a deposit of pigment. A thickening of the cuticle over the sensitive area would form a primitive lens and tend to concentrate the light upon it. From such a simple form of visual organ it is easy to elaborate gradually an eye capable of true vision.

The percipient elements of the eye are modified cells, one end of each continuous with a nerve, while the other terminates in a cuticular structure or indurated part of the cell, forming what is known as the rod or cone.

A. The **Protozoa** (unicellular) consist solely of sarcode, or animal protoplasm, displaying the essential properties of vitality, even those of movement and irritability, but they possess no organs, no distinction or separation of structures or parts.

The substance of the body is not differentiated into histogenetic elements, *i.e.* into cells or nucleated masses of protoplasm, which by their metamorphosis give rise to tissues.

The first step in the process of higher development is the conversion of more or less of the protoplasm of the ovum into such cells. These multicellular developments constitute the great division Metazoa—sub-divided into invertebrata and vertebrata.

B. **Metazoa.**—There are but few groups of the Metazoa which are not provided with optic organs of greater or less complexity.

In most instances these organs are placed on the anterior part of the brain, and are innervated from the anterior ganglia ; but in other instances they may be found situated here or there in different parts of the body. Some may permit of the *perception of objects,* while others possess a *mere perception of light.*

I.—In the **Coelenterata** some definite organisation is manifest. As their name implies, they possess " a hollow bowel," that is a distinct somatic cavity within the body with a mouth, but no formed intestine nor anus ; thus there appear two definite membranes, an ectoderm and endoderm, and muscle fibres are found in many species.

No nervous system has been differentiated, but the cells of the ectoderm may be specially modified, and give rise to nerve elements or to primitive organs of special sense. Pigment spots, eye specs, or ocelli are found in some Medusæ and Actinozoa, or short nerves distributed to pigment masses formed at the bases of the tentacles.

Thus in Lizzia a simple eye is placed at the base of a tentacle. It consists of a local thickening of the cuticle constituting a lens, and under it a percipient bulb which contains three kinds of cell elements — *pigment cells,* others suggestive of *retinal elements,* a peripheral process resembling a rod attached by a central fibre with *ganglion cells* that lie beneath it.

FIG. 1.—LIZZIA KOELLIKERI.
(From Balfour, after Hertwig.)

II.—The **Annuloida** have a complete alimentary canal shut off from the body cavity, and a primitive kind of water vascular circulation. They possess a distinct nervous system of epithelial origin usually found in the form of a ganglionated cord.

(*a*) In the *Echinodermata*, starfishes, sea cucumbers, etc., a nervous ring surrounds the gullet, and sends branches to each of the rays. The branches are probably sensory in function, and at some of their extremities is a pigment spot, which may well be a rudimentary organ of vision or ocellus.

(*b*) The *Scolecida*, or worms, show two præ-oral nerve ganglia united by a commissure, and giving off filaments in various directions. Pigment spots or rudimentary eyes, from two to sixteen, are often present, more or less close to the central ganglia.

(*c*) In the *Rotifera* there is a bilobate cerebral mass which for its proportionate size may compare with the brain of some of the higher vertebrata.

III.—**Annulosa :** Anarthropoda ; Arthropoda.

ANARTHROPADA.—In the *Annelda*, earthworms, leeches, etc., a nervous system is present, a double chain of ganglia running along the ventral surface of the body. The pair of præ-oral cerebral ganglia situated in the head (prostomium) vary in size and complexity entirely with the degree of

FIG. 2.—ANNULOSA (after Nicholson).

development of the cephalic sense organs. The cerebral ganglion is often large and complicated, but it does not control or co-ordinate the other ganglia ; its removal causes no appreciable change in the actions of the animal.

In many there is a fore and hind brain, the latter the centre of vision for the nerves to the eyes and tentacles.

The eyes of *Annelida* sometimes are mere pigment spots or ocelli placed in varying parts of the animal. Others have very perfect cephalic eyes.

In *Sagitta* they are on the head formed in a mass of pigment, with three lenses partially embedded in it, each lens associated with a rod, the retina being composed of rod-bearing cells.

The cephalic eyes of *Alciope* are highly developed, and somewhat resemble those of fish. They have a large lens adherent to the cuticle; a simple, well-pigmented retina with rods and nerve fibres connected with an expansion of the optic nerve. A well-marked fluid vitreous chamber occupies the interior of the eye and separates the lens from the retina.

ARTHROPODA.—The cerebral ganglion gradually increases in size and complexity of structure as the cephalic sense organs become more perfect, and the intelligence more pronounced. The optic ganglia become more complicated, and central nerve swellings associated with the antennæ are present. These become homologous perhaps to the olfactory lobes of vertebrates, while certain other peculiar cerebral areas are developed which may be associated with the growth of the intelligence.

FIG. 3.

(*a*) In the *Crustacea* the nervous system is of the normal homogangliate type, consisting of a longitudinal series of ganglia of different sizes united by commissural cords, and placed along the ventral surface of the body. The organs of sense consist of eyes, antennæ and in some of auditory sacs. The first pair of ganglia fuse together in a small quadrilateral body which is situated above the mouth. It gives off nerves to the eyes and to the upper antennæ. The second pair of ganglia and their commissures form a ring around the œsophagus.

In the *Lobster* the cerebral ganglion shows considerable complexity of structure. Three regions can be traced in it.

The *protocerebrum* consists of the optic ganglia and of lobes, which form a cerebral mass, partially divided by a median furrow and traversed by commissural fibres.

This complicated structure of the cerebral ganglion is apparently mainly due to its connection with the highly-developed eyes, or other less obvious sense organs, but its removal seems to destroy the capability of spontaneous locomotion, so that probably it possesses in some degree the power of controlling or perhaps even of initiating activities in the rest of the nervous system.

In the *deutocerebrum* there are two pairs of centres united by a commissure ; they probably control the first pair of antennæ, which may possess some rather complicated functions.

The *tritocerebrum* constitutes the centre for the second antennary and the tegumentary nerves.

In the *King Crab* the nervous system is concentrated around the oral end of the œsophagus into a single large ganglion, from which nerve filaments are sent to all parts of the body.

The anterior portions represent the cerebral ganglion. Anteriorly it projects as a slightly bilobed mass (the protocerebrum), from whose anterior end optic and ocellary nerves are given off to the lateral and median eyes, and to a special region in front of the mouth. There is also a great development of neurophile and ganglionic nuclei on the upper part of the protocerebrum, forming a kind of brain hemisphere, which is separated along the median line, but to which apparently no useful function can be assigned.

(*b*) (*c*) In the *Arachnida* and *Myriapoda* there is a similar concentration of the nerve ganglia around the oral end of the œsophagus. The cerebral supra-œsophageal part of the mass is bilobed, and innervates the median and lateral eyes, which are always sessile, and in the form of two to eight more or less simple eyes or ocelli.

(*d*) *Insecta.*—In these the head, thorax and abdomen are quite distinct. Five or six somites constitute the head, which possesses one pair of antennæ, a pair of eyes, and a mouth. The organs about the mouth are very complicated, and used either for mastication or suction or for both.

The nervous system consists essentially of a chain of ganglia placed vertically and united together by longitudinal cords and commissures.

The cephalic supra-œsophageal ganglia of insects are of large size. They consist of a pair of *protocerebral* lobes in connection with the optic ganglia, the size of the brain depending mainly upon the degree of development of the eyes. A second pair of centres, the *deutocerebrum*, are probably (? olfactory) in connection with the antennæ, while the *tritocerebrum* is closely associated with the sub-œsophageal ganglia by means of connectives which form a collar round the gullet, and supply the parts in the neighbourhood of the mouth.

Each *protocerebral* lobe contains a large "fungiform body," which is in close relation with other parts of the brain by means of commissural fibres, with its fellow of the opposite side, with the optic ganglia, and antennary lobes, and with a commissural band known as the "corpus centrale," to which as a nucleus fibres from all parts converge.

In spite of the evident importance of these "fungiform bodies," their function is not well known, but it is noteworthy that within the same order their size increases roughly in proportion to the intelligence of the insect; and among the several families they may even vary in development; for instance, between the persons of the society, being proportionately larger in the working bee than in the drone or queen.

In the *Hornet* the cerebral ganglia are large, but mainly because of the size of the *protocerebral* lobes in association with the great compound eyes that it possesses, and partly because of the high development of the fungiform bodies.

It is not easy to explain the very high development of communal life that exists among the higher insects, the bees, the ants and the termites, without supposing that they possess to some degree a measure of high psychical endowment. The organs of sense are the eyes and antennæ. The eyes are usually compound, and are composed of a number of hexagonal lenses united together, and each supplied with a separate nervous filament. They may be placed on peduncles, but in no case are these movably articulated to the head, as is the case in some crustaceans. In addition to the compound eyes, simple eyes, or ocelli, are often present, placed between them on the top of the head, or these latter may be the sole organs of vision, or in a few cases eyes do not exist.

THE EYES in the *Arthropoda* show several different separate types, their features of similarity perhaps depending more upon the character of the exoskeleton than on any origin from a common prototype.

There is a crystalline lens always closely adherent to the cuticle, and a retina continuous by fibres from the optic nerve, containing a layer of rods and well-marked pigment.

The percipient elements appear to be sometimes formed from the epidermis, at others from the central nervous system.

In some of the classes the eyes are *simple*, in others the eyes are *compound.*

SIMPLE EYES.

(*b*) In the *Arachnida* the organ of vision consists of from two to eight or more simple eyes or ocelli, and they are always sessile.

In *Trachearia,* the mites and ticks, which breathe by the general surface of the body or by the tracheæ, the eyes are never more than four in number.

In *Pulmonaria,* scorpions and spiders, which breathe by pulmonary sacs, the eyes are never less than six.

(*c*) The *Myriapoda* possess a variable number of simple sessile eyes behind the two antennæ. In this group are Chilopoda, carnivorous centipedes.

These simple eyes vary considerably in detail. Three types may be indicated.

FIG. 4.—CHILOPODA (after Balfour).
*Hy*—Hypodermis. *C*—Cornea.
*ON*—Optic Nerve. *R*—Retina.

1. The retinal cells lie immediately behind the lens without any intervening vitreous space or structure, as in the Tracharia.

2. The retina is separated from the lens by a distinct layer of cells from the hypodermis, which may be considered to be a solid vitreous humour. These are seen in the Arachnida, in the simple eyes of Insects, and in some Centipedes.

3. In other Centipedes and in Insect larvæ the cellular vitreous humour is formed as above from modified hypodermic cells; but the *retina seems also to be derived from the hypodermis itself*, though as is usual its outer ends terminate in rods, and their inner ends are continuous with nerve fibres which pass from the pre-œsophageal nerve ganglion.

The optic *nerve fibres* in the simple eyes of Invertebrata lie *behind* the retina, and the percipient elements (rods) are directed toward the light. But it is opposite in the Vertebrata, here the fibres from the nerve form a layer *in front* of the rods and cones.

COMPOUND EYES.

(*a*) (*d*) The *Crustacea* and adult *Insects* almost always possess compound eyes. In Crustacea the eyes may be sessile, but are usually borne on stalks attached to the first segment of the head.

In many of the larvæ there is a median eye, but after full development they are usually paired.

In the *Cyclops* and water fleas the large median eye remains permanent. In the extinct *Eurypteridæ* there was a pair of larval eyes near the centre, and also a pair of large marginal eyes in addition.

*Compound eyes* consist of an optic nerve terminated by a ganglionic swelling; this swelling has impinging on it several layers of cells, the deeper of which form a large number of elongated axial rods or "rhabdoms," the percipient elements which together constitute the structure known as the "retinula." Each rod has its own lens, which consists of a crystalline transparent cone, and on it a corneal lens facet which is developed from the cuticle.

FIG. 5.—COMPOUND EYE.
*R*—Retinulæ with rhabdoms.
*g*—Ganglion cells.

The optic nerve is connected with and is probably an outgrowth from the cephalic lobes of the pre-œsophageal ganglion, while the epidermis in the region of the future eye forms the corneal lenses, and growing columnar cells, gives rise to the crystalline cones beneath them.

Each eye forms a prominent mass on either side of the head.

The lens facets may number 50 in each eye in the case of *ants*, while in the *house fly* there may be 5,000, and in the *beetle's* eye as many as 25,000.

V. **Mollusca.**—The nervous system and the eyes vary considerably in their development.

The lower types, the *Molluscoida*, show only a single nerve ganglion. Some have no eyes, others possess pigment spots or ocelli placed between the oral tentacles or along the margins of the mantle lobes.

FIG. 6.

*A*—The *Cephalic, Cerebral, or Supra-œsophageal* ganglia. *B*—The *Infra-œsophageal ventral or pedal* ganglia. *Q*—The *Parieto-splanchnic or "Pleuro-visceral"* ganglia.

In the *Mollusca* proper the nervous system tends to be centred in three pairs of ganglia united to one another by commissures and connectives (cerebro-visceral, cerebro-pedal), which are somewhat irregularly placed.

A The *Cephalic, Cerebral,* or *Supra-œsophageal* ganglia.

B The *Infra-œsophageal ventral* or *pedal* ganglia.

Q The *Parieto-splanchnic* or "*Pleuro-visceral*" ganglia.

Nerves are given off from the cerebral ganglia to the head, to the sense organs, eyes, otocysts, tentacles and lips.

In some the two cerebral ganglia are closely paired and concentrated to form a bilateral mass of brain. The pedal and pleural ganglia are blended to form a circular pleuro-pedal mass around the œsophagus, from which centre nerves pass to the various parts of the body.

The *Encephalous Molluscs* have eyes that vary in complexity. They are present in the head, and innervated from the cerebral ganglion.

In the *Gasteropods* and others the eyes approach the vertebrate type, but are comparatively simple. A closed vesicle lined by retinal cells contains a large spherical lens which is in front in contact with the outer integument, modified to constitute the cornea.

Some snails have a very well marked-out head, a pair of eyes placed on stalks, and tentacles which may be for smell rather than tactile organs, sometimes they also have auditory capsules.

Most of the *Cephalopoda* are very highly organised. They possess considerable power of locomotion by means of the eight or ten arms that surround the central mouth. The head is very distinct, and the cerebral mass, which consists of the supra- and infra-œsophageal ganglia, is protected by a cartilaginous case or kind of rudimentary cranium. The most highly organised invertebrate eye is that of the Dibranchiate Cephalopods.

FIG. 7.

*R*—Retina. *C*—Cornea.
*g*—Ganglion. *ON*—Optic Nerve.

In the Cuttle-fish there is a kind of *orbit*, which contains the *optic ganglion* into which the *optic nerve* passes. The eye possesses a modified *sclerotic*, a *choroid*, *iris* and *ciliary muscle*, a *lens* and *vitreous humour*.

The *retina is complicated*. In front are *rods* and *granules* imbedded in *pigment*, posteriorly is a layer of *nerve fibres*

separated from the pigment by cells and connective tissue.

A *cornea* formed of cuticle may enclose the eye in front, or there may be an opening through it leading into the "anterior optic chamber," which is large and complicated and homologous to the aqueous.

### VERTEBRATA.

One of the most obvious, as it is most fundamental, of the distinctive characters of the Vertebrata is to be found in the shutting-off of the main masses of the nervous system from the general cavity of the body. As in the sub-kingdom Annulosa, so in the Vertebrata, the body is composed of a number of definite segments arranged along a longitudinal axis.

But in all Invertebrate animals without exception the body may be regarded as a *single tube*, enclosing all the viscera and the nervous system also within the general cavity of the body.

In most invertebrate animals the lines of the nervous system are perforated at the gullet, so that an œsophageal nerve collar is formed. Almost all of the nerves and commissural bands are ventral or post-œsophageal, but there is nearly always one well-defined prœ-cesophageal ganglion which is the homologue of the vertebrate brain, though its functions are very limited, and are analogous probably to those of the mid-brain and thalamencephalon, their association with sight being perhaps the most important.

In the Vertebrata a *special tube* contains the cerebro-spinal centres, to separate them from the rest of the body. It is formed along the middle line by the appearance of the "primitive groove," with a rising above along each side, the "laminæ dorsales," which become more and more raised up till they meet in the middle line, and form the tube within which the cerebrospinal nervous centres are developed. By this means the main masses of the nervous system are entirely shut off in a special tube from the rest of the body and superadded to the ventral cavity, which contains, both in Vertebrates and in Invertebrates, the alimentary canal, the hæmal system and the ganglionic or sympathetic nervous system.

Moreover, the floor of the primitive groove becomes developed into the chorda dorsalis or notochord. This remains persistent throughout life in the Lancelet and a few others, but almost universally gives rise to the vertebral column, the essential feature of vertebrate animals.

The *brain is developed* from the expanded upper end of the medullary tube. The primitive division of the nervous axis is probably not into brain and spinal cord, but the fore-brain is first separated from the parts behind it, the mid and hind-brain and the spinal cord.

FIG. 8.
EMBRYO CHICK, THIRD DAY.
(After Balfour.)

*A*—Prosencephalon. *B*—Medulla oblongata. *Cr*—Cerebellum. *Ch*—Choroidal Fissure. *M*—Mid-brain. *O*—Optic Visicle. *Th*—Thalamencephalon. *V*—Visceral Folds. *k*—Auditory Vesicle. *n*—Nasal Pit.

In the embryo the length of the brain is very much greater than that of the spinal cord.

The fore-brain is histologically very distinct from the other parts, and the nerves coming from it have a different character from those of the mid and hind-brain. Moreover, the front end of the notochord is opposite the front of the mid-brain, in a line which separates the fore-brain with the thalamencephalon from the mid-brain. The mid-brain lies *on* the notochord, it is the homologue of the optic lobes, and contains the ganglia of the oculo-motor nerves. The *mid-brain* remains single, while the hind-brain is a more complex structure giving rise to the "cerebellum" and "medulla," from which arise a large number of segmental nerves which supply the regions of the face and mouth.

The *fore-brain,* divided into "thalamencephalon" and "cerebral hemispheres," becomes highly developed in man and higher mammals in regard to some extent to the area of the surface or to the size of the body, but chiefly in accordance with the greater necessities and higher intellectual development of the individual.

I. **Fishes.**—The Lancelet has a persistent notochord. A brain is scarcely present, but the fore part of the nervous axis gives off nerves to a pair of very rudimentary eyes.

(*a*) In *Cyclostomi,* lower fishes, Lampreys and Hag Fishes, the brain is extremely simple, it may be said to remain in an embryonic condition. The individual vesicles lie in a line horizontally behind one another (? persistent notochord).

The "medulla" forms nearly half the brain, and from its roof arise the usual cranial nerves.

The "cerebellum" is a mere narrow bridge in front of the "Rhomboid Fossa." The mid-brain is relatively broad; it consists of a pair of prominent rounded eminences, the optic lobes, which from their outer surface give rise to the optic nerves. A band of fibres, the posterior commissure, marks the boundary between the mid-brain and the thalamencephalon; the latter is deep, and shows on its posterior surface gangula habenulæ in contact with pineal body (epiphysis), its inferior surface or floor is expanded down to form a capacious infundibulum. The sides are covered by the optic tracts, which pass downward and forward from the optic lobes to the chiasma in front of the infundibulum.

The thalamencephalon is continuous anteriorly with a pair of lobes (the fore-brain or prosencephalon), from which the olfactory lobes open out.

(*b*) The *Elasmobranchii*, or cartilaginous fishes of Cuvier, show a skull and lower jaw well developed, but there are no cranial bones, and the skull consists of a cartilaginous box without sutures.

The nervous system and cerebral mass is more highly developed proportionately than is the case with any other division of the fishes.

The " thalamencephalon " is somewhat narrow, the optic lobe small compared with the Teleostan group. The " cerebellum " very largely developed, overlapping the " optic lobes " and " medulla." There is great development of the olfactory bulbs and peduncles very large and solid.

All the organs of sense are in a very high grade of specialisation. This order contains the shark, skates, rays and the dogfish, in some of which the visual organs and optic lobes are very strongly developed, in others weak.

(*c*) In the order *Teleostei*, which includes the great majority of fishes, the bony fishes of Cuvier, the skull is of an extremely complicated nature, being composed of a number of distinct cranial bones, and a mandible and lower jaw is invariably present.

The floor of the " prosencephalon " shows well-marked swellings or corpora striata connected by an anterior commissure, and covered over by a thin epithelial pallium.

The brain is smaller, and the olfactory portion unimportant. The " thalamencephalon " is very small and depressed between the mid and fore-brain ; there is a marked " epiphysis," the " infundibulum " and " hypophysis " with "saccus vasculosus" are strongly developed with lobi inferiores and a pituitary body.

The mid-brain is extremely large, the optic lobes are very strongly developed, and the nerves that supply the eyes are well marked, while the olfactory region is peculiarly feeble.

As in all other fishes, the optic nerve crosses completely over to the opposite lobe. The nerves may be free at the chiasma, crossing one above the other, or they may interlace without association. The cerebellum is well marked.

The cod, brill, roach, salmon and carp are members of this group, and in detail different parts may vary greatly.

In the *Sole* the skull is twisted so that the two eyes lie on one side of the body, as the fins show, usually on the right. The optic nerves are equal in size.

"The left nerve passes above the right and twists round above the sphenoid bone to reach the left eye, which is displaced to the right side of the fish."

THE EYE IN FISHES has to be modified in accordance with the great density of the water medium through which the rays of light pass. The cornea being scarcely curved, assists but little in refraction; but this is compensated by the lens, it is a dense spherical body with a very high magnifying power. The retina is approximated to it by the flattened eyeball.

FIG. 9.

The aqueous humour scarcely exists, and the vitreous is small in extent. To protect the eye from varying pressure, from injuries and stormy seas, the sclerotic is strengthened by cartilaginous plates, or sometimes by a bony cup. The accommodation, which is limited in extent, takes place in Teleostii by means of a process of the choroid, the "processus falciformis," which is attached to the lens by the "campanula Halleri." This structure contains nerve, vessels and smooth muscle fibres, which probably draw the lens towards the retina. It is the homologue of the pecten found in birds and reptiles.

II. **Amphibia.**—The brain on the whole is less complicated than that of any other Vertebrates, in some it is of very low type.

The hemispheres are cylindrical, and separated as far back as the anterior commissure, and connected to the simple thalamencephalon by a large basal tract. The olfactory regions are connected by a hippocampal commissure.

In *Urodela* the optic lobes are very weak, and in the Proteus Anguinus, which spends its existence in dark caves, it would seem that vision is altogether absent, and as might be expected, the optic lobes can scarcely be recognised. But in the Frog the optic lobes form the broadest part of the brain.

III. **Reptilia.**—The brain reaches a considerably higher stage of development. For the first time there is an undoubted cerebral cortex. The pallium is much larger than in an Amphibian, it is more highly developed,and also more highly differentiated. Some show a distinct hippocampal formation witha fornix. The olfactory bulband tract are well shown, or not, according to the degree of smell required by the individual.

FIG. 10.

*A*—Prosencephalon. *B*—Medulla oblongata. *Cr*—Cerebellum. *E.P.*—Pineal Eye. *M*—Mid-brain. *Th*—Thalamencephalon. *Epi*—Epiphysis. *Hypo*—Hypophysis Infundibulum. *Olf*—Olfactory.

The cerebellum is small, excepting in the Crocodile. The mid-brain has two well-marked optic lobes, showing a tendency to sub-division into four, from which the optic tracts pass forward to the chiasma.

The "thalamencephalon" is hidden between the "prosencephalon" and "mid-brain." The *epithalamus* is well developed, especially in Hatteria, where is shown a well-marked pineal body with a parietal eye passing forward in contact with it. This eye arises independently from the brain above, from whence a rudimentary nerve passes into a pigmented retina, which is contained in a vesicle, the dorsal wall of which is thickened to form a lens. The vesicle, which may be looked on as an intraocular or vitreous fluid, is contained in a thickened capsule, the front of which is clear, like a kind of cornea. This eye is situated in the parietal foramen of the skull in Hatteria, and a similar structure is found among Lizards and in the embryo of the Vipers.

In many Reptiles the covering of the eyeball is peculiar. Lizards, Crocodiles, etc., possess eyelids, but Snakes and Reptiles do not; they show instead a mere scaly ridge surrounding the surface of the eye, which is itself covered by a layer of transparent modified epidermis. Under this lies a close, flattened conjunctival sac containing lacrymal fluid.

The epidermic or "autocular membrane" over the eye is shed together with the scaly skin, so that for some days the Snake is blind. The prominent large eye of the Chameleon is covered by the two lids, which coalesce to form a single circular membrane, in which is a small central palpebral aperture through which the eye can see.

IV. **Aves.**—In *Birds* the brain is remarkably constant in form. The cerebrum, optic lobes and cerebellum are large and wide. The cerebrum contains highly-developed corpora striata, and there are well-marked *pseudo-occipital lobes* and smaller pseudo-temporal lobes. The olfactory bulbs are weak. The optic lobes and thalamus are very well developed; and there are strong and numerous fibres forming the *cortico-thalamic tracts* passing to the pseudo-occipital lobes, to the tectum opticum. The geniculate bodies are enormously developed. The optic tracts form a complete chiasma in front of the infundibulum.

FIG. 11.

OWL'S BRAIN. ANTERIOR ASPECT. (After Wiedersheim.)
*A*—Prosencephalon. *B*—Medulla Oblongata. *M*—Mid-brain. *Th*—Thalamencephalon. *Hypo*—Hypophysis Infundibulum.

The thalamencephalon shows two rounded eminences, optic thalami, covered laterally by the optic tracts. All the nerve fibres pass in bundles through the chiasma to the opposite side of the brain; but in the Owl, with its strongly developed optic lobes and thalami, a few fibres are said to pass direct from each optic nerve to the tract of the same side, to allow of some binocular vision.

On the third day in the embryo chick the choroidal fissure near the optic nerve admits into the eye a vascular loop—this helps to form the vitreous humour. Later on it forms the pecten, which consists of fine blood vessels in connective tissue with a main artery and vein, it probably nourishes the contents of the eyeball, but is not concerned in accommodation.

FIG. 12.

IN BIRDS the cornea is very prominent, with a deep aqueous chamber. The antero-posterior diameter of the eyeball can be elongated by means of bony plates in the sclerotic, which extend back from the margin of the cornea. These when compressed overlap, lengthen the eyeball and protrude the cornea, thus preparing it for near vision; while relaxation of the muscles allows of recession, flattening of the cornea, and fits the eye for distant sight.

The iris of birds is very pigmented, and is active in contraction of the pupil, and perhaps in accommodation.

In Mammals the sclerotic is never (?) supported by a ring of bony plates as in birds and many reptiles. But the eyes of Cetacea and of some amphibious Carnivora are built on a similar type to the eyes of fishes.

V.—The **Mammalia** are divided into *Placentals* and *Non-placentals*, and were grouped by Professor Owen into four classes according to the development of the structures of the brain.

(*a*) 1. *Lyencephala.*—Loose brained. The olfactory and optic lobes and the cerebellum are not covered in by the cerebral vesicles.

This class comprises the Non-placental Mammals, Monotremata and Marsupials. There is a large cerebral cortex which is smooth, and not very definitely linked up with other parts of the nervous system, few associated nerves coming from it. There is no corpus callosum, but the hippocampus is highly developed, with its dorsal and ventral (supra and proe) commissures, and is very characteristic of the brain in Marsupials and Monotremata.

*Monotremata.*—In the *Platypus* there is a poor visual apparatus. The optic and oculo-motor nerves and associated organs are small. Many of the animals are much in burrows, and the sense of sight is unimportant; smell is also unimportant; the olfactory bulb and tubercle and the pyriform are somewhat small. The *snout*, however, is very large, and lined by special endings of the fifth nerve, which are enormously developed with the sense of touch.

In the *Spiny Ant-eater* the snout is large, but the fifth nerve unimportant. There is, however, *great development in the olfactory region,* and the rhinal fissure is very deep.

The optic thalami and mid-brain are well developed, but the geniculate bodies are absent, and the corpora quadrigemina weak, while the eyes and optic tracts are very small. There is no calcarine or splenial fissure in the Monotremata.

In the *Marsupials* the calcarine fissure appears, running obliquely above and behind the hippocampus. The occipital region is as yet unimportant. This splenial or calcarine sulcus is an early developed and important centre for vision; it is absent or weak in the Wombat (Marsupials) and in most Rodents, but otherwise it is almost universally found as a definite feature in Marsupials and Placental Mammals.

FIG. 13.
MEDIAN SURFACE OF BRAIN.
*a*—Calcarine Fissure.

In Marsupials the Tasmanian wolf Thylacinus has a well-marked optic tract, corpora quadrigemina, and geniculate bodies attached to the optic thalamus.

(*b*) All *Placental Mammals* possess a corpus callosum. As the cortical areas of the brain increase in size this strong commissure becomes necessary, and it develops further with the importance of the hemispheres. With a stronger callosum the hippocampus diminishes, its anterior or cephalic extremity becoming weak and vestigial, while the posterior end continues to be large and important, even forming the hippocampus major of the lateral ventricle in man. Compare Figs. 40, 55, 76, *Royal College of Surgeons' Catalogue of Museum,* Physiological Series, vol. 2.

### ERRATUM.

FIG 15.—Read MEDIAN "SECTION" *for* "SURFACE."

2. *Lissencephala.*—Smooth-brained, very few convolutions, cerebellum and olfactory lobes exposed.

Cheiroptera, Insectivora, Rodents, Edentata.

3. *Gyrencephala.*—Many convolutions, olfactory and cerebellum much covered.

Cetacea, Sirenia, Ungulata, Carnivora, Quadrumana.

4. *Archencephala.*—Overlapping brain. Man.

*Insectivora,* usually nocturnal and subterranean. The simple brain of the Hedgehog closely resembles that in Marsupials, there is an extraordinary development of the olfactory and of the pyriform lobes. Here, as also in the Mole, as might be expected in a blind animal, the optic parts of the brain are poorly developed. These parts can, however, be well recognised in the young mole, which possesses some power of vision.

In the *Galeopithecus,* Flying Lemur, the anterior quadrigeminal bodies are extremely large, bulging up between

FIG. 14.
MEDIAN SURFACE OF BRAIN.
*a*—Calcarine Fissure.

FIG. 15.
MEDIAN SURFACE OF BRAIN.
*Cr*—Cerebellum. *M*—Mid-brain.
*Th*—Thalamencephalon.

the cerebrum and cerebellum, and there is a large horizontal calcarine fissure.

*Cheiroptera* (Bats).—The organs of the brain are simple, and similar to those of the family above, the eyes are small, but the calcarine and intercalary sulci are well developed.

The *Rodents* have a large brain well supplied with blood, showing but few convolutions ; possibly its large surface is sufficient for the needs of the members of the order without any increase in the surface area, which is produced by the presence of sulci. These are practically absent, especially on the mesial surface, where even the calcarine sulcus is absent or insignificant. Yet the Beavers show much intelligence and ingenuity in their communal life.

In the Squirrels the optic nerve is large, and the anterior quadrigeminal bodies highly developed, though they are hidden by the large cerebral hemispheres. In the Rodents the orbit communicates by an opening in the septum, and forms a common cavity with the temporal fossa. The eyes are directed laterally outwards, and can move as easily backward as forward, as if to provide for escape rather than attack, and they have no sort of binocular vision.

In all Fish, Amphibia, Reptiles and Birds, with perhaps exception of the Owl and a few birds of prey, all the optic nerve fibres decussate from the retina of either eye to the opposite optic lobe. In the exceptions given a few direct fibres pass to the same side of the brain.

*Edentata,* one of the lowest orders, show considerable development of the occipital region of the brain with well-marked sulci.

FIG. 16.—MEDIAN SURFACE OF BRAIN.
*a*—Calcarine Fissure. *b*—Intercalary.

The Armadilloes and Aardvark show a well-developed "complex splenial" sulcus, representing the calcarine, intercalary and genual, as is usually found in the Mammalia. The Sloths and Ant-eaters show a vertical calcarine separate from the intercalary, as occurs in Primates and in Lions. The Pangolin also shows a separate vertical calcarine, and a retro-calcarine pushed back separately into the occipital lobe by the increase in the parietal and other parts of the brain hemispheres.

In the *Carnivora* the brain power is highly developed. The hemispheres pass forward over the olfactory bulb, and backward over the cerebellum.

Hearing is very acute, and we find a large mesial geniculate body, and large posterior corpora quadrigemina. In the fruit-eating Carnivora the eyes are at the side of the head, and they possess only a limited convergence, but in the *Felidæ* and others the eyes are set forward, and the pupil is very active, and there is some degree of binocular vision.

These animals require good distant vision, often when the light is dull, and they need also very reliable closer sight, and a most perfect co-ordination of the eyes with the fore limb by which they catch their prey. In the optic chiasma there are present considerable direct as well as decussating fibres.

FIG. 17.—MEDIAN SURFACE OF BRAIN.
*a*—Calcarine Fissure. *b*—Intercalary. *c*—Retro-calcarine

A sylvian fissure is well developed, especially in the Lion. On the mesial surface, there is a deep calcarine sulcus below, which joins the intercalary and passes up towards a crucial sulcus in the middle line, shutting off a posterior lobe from the rest of the brain, which lobe, I assume, is concerned in vision. The occipital lobe may be very well developed.

In some species there appear secondary fissures running out of the calcarine, or even a definite retro calcarine, or collateral, besides other associated sulci are found varying in size.

The *Ungulata* are large animals, and they need a large brain surface and many convolutions, but the mesial area is very simple, not dissimilar to that of the Ant-eater.

There is no high specialisation in the calcarine fissure, it is large and placed behind the splenium. It joins the intercalary, and that the genual, showing a complex splenial.

The eyes are usually placed on the side of the head, and separated by the forehead or nose, there is a wide area of *periscopic sight,* but they have only a limited amount of convergence and binocular vision. The Deer are particularly helped by a high degree of smell and hearing. In the Horse about one-sixth of the fibres do not decussate in the chiasma but pass direct.

In *periscopic vision* each eye is responsible for the field on its own side, and as this is represented on the opposite side of the brain, *all* the optic nerve fibres must decussate. When the eyes tend to converge and give slight binocular vision a part of the nasal side of each field is overlapped, the superimposed parts have crossed to the opposite side of the middle line. The extreme nasal field of the right eye is now concerned with the left field of vision, and the fibres that represent this must go to the opposite side of the brain; thus we have some fibres of the extreme temporal side of the retina of the right eye passing direct to the right side of the brain. As the eyes turn more forward, more and more of the nasal fields overlap, and more and more direct fibres are required. As binocular vision becomes more perfect the lateral range of sight is lessened. In perfect stereoscopic vision both visual axes must be turned towards the object looked at. The whole of each nasal field is carried across the middle line, and is superimposed upon the temporal field of the opposite side. We thus have an almost complete overlapping, only the extreme temporal areas remaining single. But far more important is the overlapping of the maculæ.

The acuity of the macula depends on the delicate association of each cone (7,000 or 8,000 in number) with bipolar nerves and ganglion relay centres. Both halves of each macular bundle of nerve fibres probably pass along the decussating and direct portion of both optic tracts, and are probably distributed widely over the visual cortex of both cerebral hemispheres. The macular area in the cortex is probably widely distributed, or if it is localised, it is especially well supplied with blood with a free anastomosis, thus giving it great resisting and recuperative power.

Lindsay Johnson says that a true macula is only found in men and the monkeys, and they alone probably possess parallel vision with complete convergence.

In the Monkeys the calcarine sulcus becomes the centre of further developments, it is quite separated from the

FIG. 18.—MEDIAN SURFACE OF BRAIN.

*a*—Calcarine Fissure. *b*—Intercalary. *c*—Retro-calcarine. *d*—Collateral. *e*—Parieto-occipital. *f*—Intraparietal.

intercalary, which with the rostral becomes the calloso-marginal. In the Aye-Aye and Lemurs it is somewhat vertical, but in the Tamarin, one of the anthropoid apes, and Marmoset a long single sulcus is prolonged horizontally far back into an elongated occipital lobe which measures nearly half of the brain.

In the Squirrel Monkeys (Cebidæ) almost half the hemisphere lies behind the splenium. The calcarine sulcus terminates in a wide-shaped bifurcation, and several other compensatory calcarine sulci are developed. The collateral runs forward on its ventral side, and from its dorsal runs up a parieto-occipital sulcus close to but behind the intraparietal. The intraparietal in man is the only sulcus of the parietal lobe, it lies behind the central sulcus, and has the following divisions: (*a*) post-central superior, and (*b*) inferior, (*c*) horizontal, (*d*) occipital.

The frontal lobe is separated from the parietal by the fissure of Rolando.

In the Macacus we have the higher organisation of the old world Monkeys (Cercopithecidæ). The smell organs are well seen, but diminishing. The long bifid retrocalcarine, the collosal and the parieto-occipital, with the intraparietal sulcus, are well developed. This brain shows the same sulci as Fig. 226, and also the usual human structures—corpus collosum, fornix, anterior commissure, etc.

The axis processes from the optic tracts and lateral geniculate bodies traverse the posterior end of the internal capsule at the junction of its superior and inferior laminæ behind the lenticular body, and then pass directly backward as the *optic radiations* towards the occipital lobe. They run along the outer wall, roof and floor of the posterior horn of the lateral ventricle, and end in the nervous felt work of the occipital cortex along the calcarine fissure.

The occipito-thalamic radiations consist chiefly of "corticipital fibres" for sight, but corticifugal fibres also pass along them to the superior brachium and quadrigeminal colliculus, and thence to the oculo motor nerves.

The visual path has its anterior neurons running from the retinal elements to arborize in the cells of the external geniculate bodies, and from them the posterior neurons run on to nerve cells in the occipital cortex in and around the calcarine fissure. It is therefore not the retinal fibres, but the fibres of the external geniculate body that are projected upon the occipital lobe.

### THE VISUAL CORTEX IN MAN.

The *calcarine fissure,* which in man commences a short distance behind and below the splenium, was first well described by Cunningham.

FIG. 19.*—MEDIAN SURFACE OF BRAIN—MAN.

*a*—Calcarine Fissure. *c*—Retro-calcarine. *d*—Collateral. *e*—Parieto-occipital. *m*—Sulcus Limitans, superior. *n*—Sulcus Limitans, inferior.

It consists of an anterior part, or "stem," the "true calcarine fissure," which protrudes into the posterior horn of the lateral ventricle as the "calcar avis."

* Fig. 19. Specially drawn from a brain in Royal College of Surgeons' Museum, and kindly approved by Professors Keith and Elliot Smith, to whose work I am further indebted in this paper.

Other diagrams are copied or modified from the *Catalogue* (*Physiology*) *of the Royal College of Surgeons' Museum,* from Balfour's *Comparative Embryology,* and from Wiedersheim's *Comparative Anatomy,* and I beg to express my obligations.

The fissure as it passes backwards appears to bifurcate into the posterior or retro-calcarine, and the occipito-parietal fissures. Really, however, the latter is separated from it by the *annectant cuneal gyrus*, and the former by the *deep anterior annectant cuneo-lingual gyrus*.

The *posterior calcarine* itself also seems to bifurcate behind into an upper and lower vertical fissure, the "fissura extrema" or "terminalis," but it is really separated from them by the posterior annectant cuneo-lingual gyrus.

If sections are made through the cortex of the occipital lobe, there is seen running across the calcarine fissures and parallel to the grey matter which covers the surface of the lobe a well-marked, easily-seen white line. This is continuous with and is, as it were, an exaggeration of the line of Baillarger, which can be seen in many parts of the grey matter of the brain, especially in the superior parietal lobule.

In the occipital lobe the white lamina is especially easily seen on section, and is known as the line of Gennari. The line is due to the presence of a special plexus of nerve fibres, mostly delicate, but many of stronger calibre, and some very stout fibres running in the cortex and traceable for a considerable distance; within its deeper surface are the endings of the optic radiations.

Elliot-Smith has most carefully described its distributions. It stops abruptly, and can be readily traced, and the area over which it passes can be easily identified. It runs in the grey substance of the occipital lobe following the sulci and convolutions, and by means of its presence the area concerned in vision can be accurately mapped out. Though it appears as a line in sections of the brain, it is really, of course, a layer of special tissue, which forms part of the thickness of the cerebral grey matter, and lies halfway between the surface and the underlying white matter of the brain.

At the retro-calcarine fissure it is seen to line the hollow and both sides of the sulcus, and it reaches *upward* on the cuneal gyrus, and *downward* on the lingual gyrus, as far as the two small sulci, which run nearly parallel to the retro-calcarine, namely the "sulcus limitans superior" *above*, and the "sulcus limitans inferior" *below*; these meet together in the pole of the occipital lobe, and bound the area striata at that part. It is only prolonged a little to the posterior lateral surface of the hemisphere around the tip of the occipital lobe. Here it is strictly bounded by the sulcus lunatus, which is itself free of the striate tissue.

The area of cortex which it involves is called by Elliot-Smith the "area striata." This is the "visuo-sensory area" of Bolton, Campbell and others, the "primordial visual area" of Fleshing.

**The Visuo-sensory Area.**—The structures concerned have been most carefully examined by Campbell, who finds that the special lamination included in the line of Gennari shows its largest dimensions at the forked termination of the posterior calcarine fissure which it surrounds, and just reaches the pole of the occipital lobe; and as the length of one or other limb of the fork may be greater or less, the extent of the striate tissue will also increase or diminish, not only in length but in breadth and substance.

It spreads forward, bounding the retro-calcarine above and below, involving the cuneo-lingual gyri at either end of that fissure. It spreads definitely below, occupying the lingual gyrus, and passes forward half-way along the lower border of the true calcarine fissure. Above it is well marked at the back of the cuneus; but anteriorly, at the angle where the occipito-parietal sulcus leaves the calcarine, it has gradually ceased to exist, and it is not found in the cuneal portion of the annectant gyrus at that part.

The occipito-parietal sulcus is quite free of it, so is the gyrus fornicatus. There is no striate tissue along the upper margin of the calcarine proper. Both Campbell and Elliot-Smith insist that it is only found along the lower margin. On the other hand, the retrocalcarine is extensively and completely surrounded by the stria Gennari both in man and the anthropoid apes, Elliot-Smith proposes to call it the "medial intra-striate sulcus."

Microscopic examination shows that the line of Gennari is composed of a dense network of fibres, in which terminate the fibres of the optic radiation in a pale-stained area. The cells that are distinctive of this region are curious large triangular or quadrilateral-shaped stellate cells, found chiefly along the calcarine fissure, and in the deeper parts is a layer of pyramidal giant cells, the solitary cells of Meynert.

**Visuo-psychic Area.**—Outside the visuo-sensory area is a border about two centimetres in width, the *visuo-psychic area*. It covers the cuneus above, but not the occipito-parietal fissure, nor the upper edge of the true calcarine, but it occupies its lower border almost in its whole length. It passes over the back part of the collateral fissure and round the occipital lobe, rejoining the cuneus above.

The gyrus fornicatus does not seem to be a part of the substratum of vision.

The *visuo-psychic* region has no line of Gennari, but there are a very great number both of cells and of fine fibres showing a high degree of functional activity. The peculiar stellate cells have disappeared, and there are no large cells of Meynert; but on the other hand there are many large pyramids, some of which are developed into giant cells, characteristic of this layer, with several roots below, and one very long-drawn-out process above.

This area probably transfers impulses received from the "visuo-sensory" to further districts in the brain associated with the sense of sight.

Dr. Mott, in his interesting Bowman Lecture on the progressive development of the visual cortex, shows how the width and complication in structure of the cortex gradually increases as we ascend the animal series, and how the special cells become more necessary.

Thus in the *Hedgehog*, in addition to polymorph and small stellate cells, are found occasional large pyramids. In the *Rabbit* there is a line of Gennari, large stellate cells and branching pyramids.

*Ungulates* have a well-marked line of Gennari, and numerous solitary cells of Meynert. In the *Cat* there are numerous cells of Meynert, but the most striking feature is the depth of the pyramidal layer ; these pyramids are also well developed in the cervical region of the cord, and Mott suggests they lie along the volitional path of the executive faculty, which is exercised through the fore limb and with the help of binocular vision.

The depth of the pyramidal layer increases after birth. Dr. Mott considers that the progressive development of the pyramidal layer in the visual cortex of Mammals is associated with an increase in the perfection of binocular vision.

Flesching has shown, by preparations of the fœtal brain, how the development of different parts of the visual areas takes place.

He considers that as there are many degrees of functions, so the nerve fibres develop as they are required—the *sensory* found at birth, the *motor* found soon after birth, and the *associated* which gradually develop.

Thus at birth only a portion of the fibres in the optic-thalamic radiations have acquired their *myelin investment.* All those that have done so come from the lateral geniculate bodies, and they go direct to the calcarine fissure. Those fibres that come from the pulvinar appear to be medullated later in life, and to pass outside the immediate limits of the calcarine area.

Medullation of the fibres in the cortical areas occurs at different times. Thus an infant very soon sees light, but does not show that it sees an object held in front of it for some weeks, while it takes him as many months to turn his eyes to follow an object held at his side, or to stretch out his hand to get it.

It is obvious that the highly co-ordinated work that depends on sight is constantly needing the development of new cells and of association fibres throughout life, and they are far more easily acquired in early life.

**Visuo-sensory Association Fibres.**—The visuo-sensory area along the calcarine fissure is the primary station in each hemisphere for the reception of impressions coming from the retina through the geniculate bodies; around it is the visuo-psychicarea to which these impressions are transferred; this occupies the rest of the surface of the occipital lobe, its functions are to elaborate and to interpret. If part of the visuo-psychic area is primarily diseased, there is likely to be a partial hemianopsia complicated by slight peculiarities of vision more or less indefinite, some difficulty in memory of words, some form of letter, word or mind blindness.

When the fibres that go to the temporal region are affected there may be word deafness, or loss of power of the musical faculty, to recall names, or to read aloud.

The psychic cells are associated with the "psychic motor," where the impressions or information gained by sight are transferred for purposes of thought, speech or action, and these with the "emisive motor" by which speech and writing are effected; the centres for these latter acts lie adjoining those for the simple movement of the lips and hand.

The various parts of the visual cortex are closely connected by short association fibres almost infinite in their distributions, and more distantly by long association fibres, many of which are collected in well-defined bundles.

Adjoining the occipital lobe, and continuous with its lateral gyrus, is the angular gyrus, which appears to be a higher visual centre of some importance, probably developed mainly on the left hemisphere.

The angular gyrus might be readily associated with the primary visual area by means of fibres running through the occipital lobe, or with the psychic area, or with the pulvinar or elsewhere by association fibres.

The central sulcus of each hemisphere separates the motor area (frontal) in front from the sensory area (parietal) behind. The interparietal sulcus runs upward almost parallel behind the central; it then turns horizontally backwards parallel to the upper margin of the hemisphere and terminates in the occipital lobe behind.

It separates the post-central region of common sensation (and the superior parietal occipital gyri) from the supra-marginal and angular gyri. These two latter are probably the centres for the recognition of higher, more elaborate forms of sensation and of sight and hearing.

The pupil reflex passes through the lower centres, corpora quadrigemina and third nerve, it is quite unconscious, and does not reach the cortex of the brain.

But pupil movements are also controlled by impulses centrifugal from the visual cortex through the brachium superius and the quadrigemina.

Many of the simpler and most constant movements that occur in association with sight are automatic, practically reflex, in such involuntary movements as looking towards a sudden light or to moving objects. Wide movements of the head and limbs are constantly necessary in avoidance of obstacles, and express visual reflexes of varying complexity. For them associated fibres run from the visual cortex to the motor centres from which the movements emanate: some of these tracts are in such constant co-operation that the associated movements become a kind of compound reflex action gradually evolved by constant use.

Eye movements intimately associated with the most complex mental activities are most of them involuntary and unconscious; consciousness is alone concerned with the result effected.

The visual centres are undoubtedly reached by other afferent impulses than through the eyeball and retina alone. Muscle sense produced by pupil action and accommodation, and movements of the eyes in any direction, or in convergence, cause simple impulses towards the brain. Visual judgments also largely depend on impressions from the sense of touch, and as almost every movement of the body is guided by sight, the associations around sight are very wide indeed.

When the sight is lost from defects in the *eyeball or optic nerve* almost all other impulses from hearing, touch, muscle balance, etc., are effectual in stimulating a healthy visual cortex, and they become increased in influence to the help of faulty sight. But *defect in the visual brain* is a much more serious matter, and if the sensory area is damaged by disease some part of the psychic area is likely to be affected with it.

## Figures and Tables

**Fig. 1. f1:**
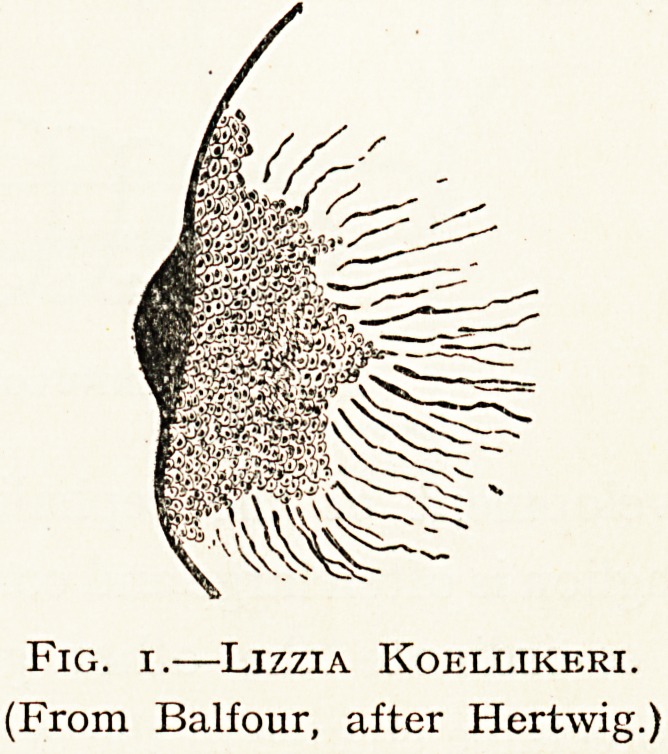


**Fig. 2. f2:**
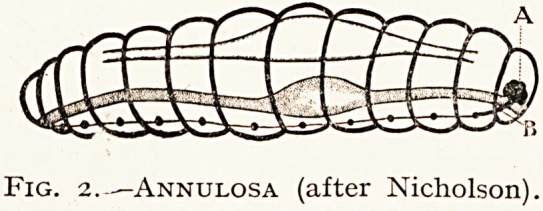


**Fig. 3. f3:**
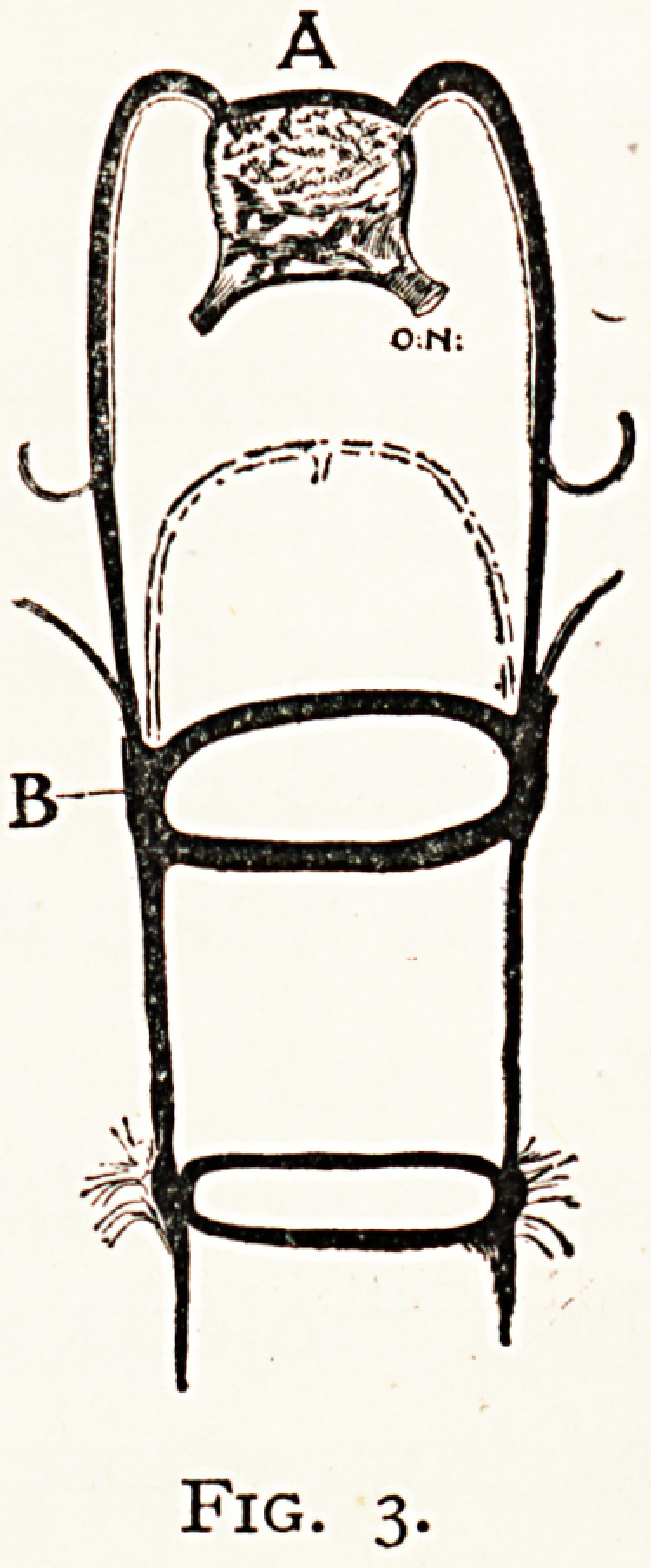


**Fig. 4. f4:**
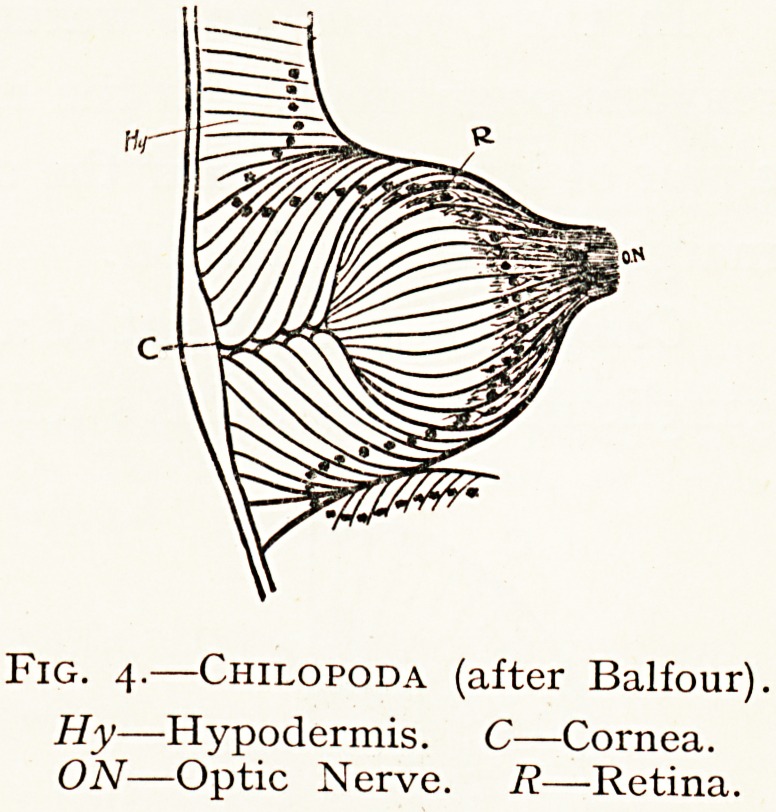


**Fig. 5. f5:**
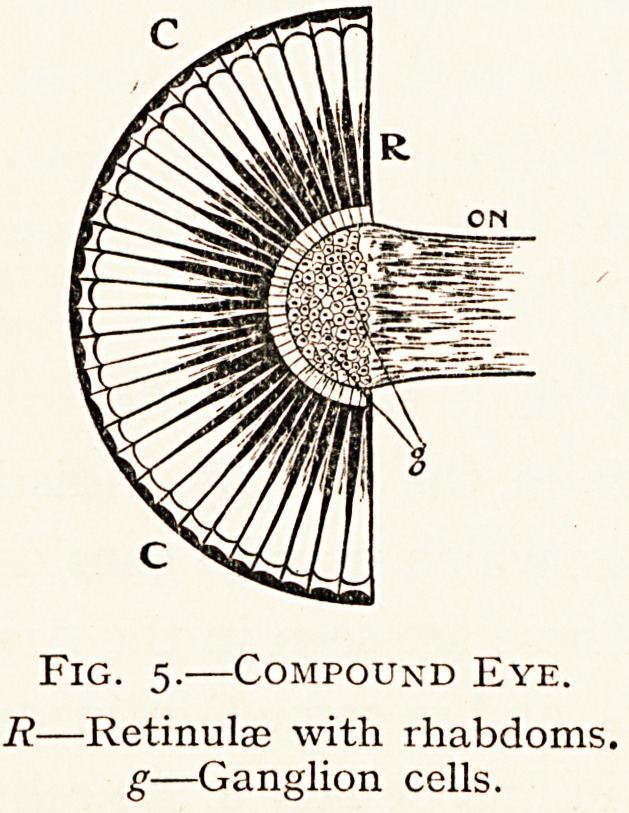


**Fig. 6. f6:**
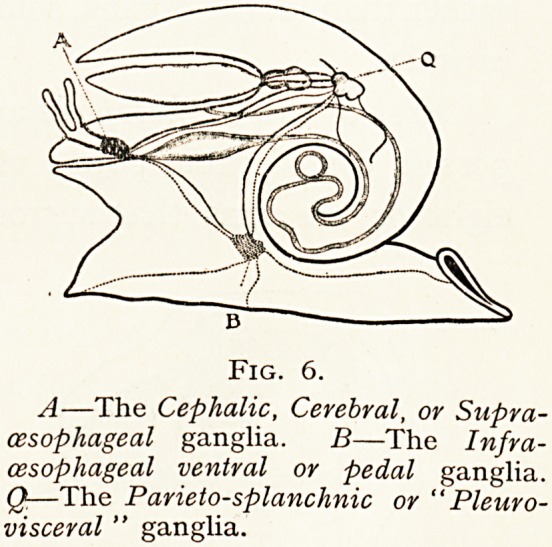


**Fig. 7. f7:**
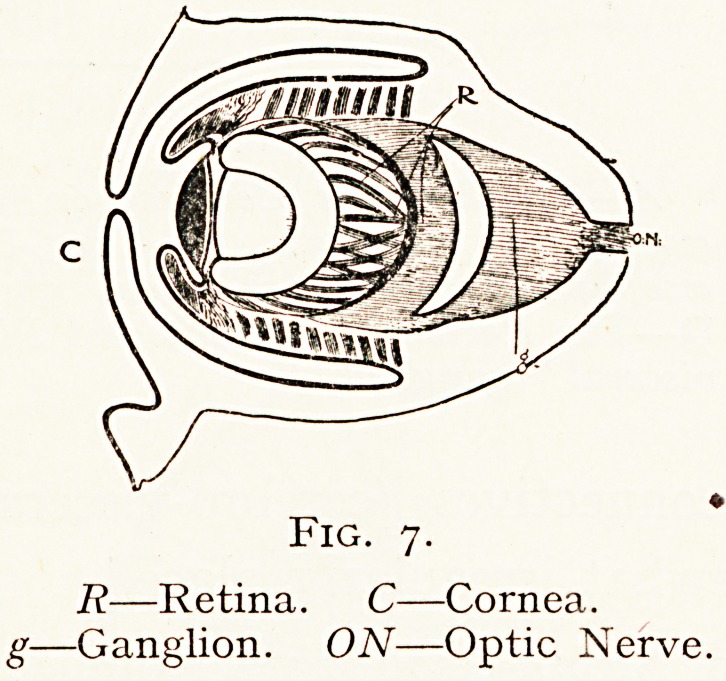


**Fig. 8. f8:**
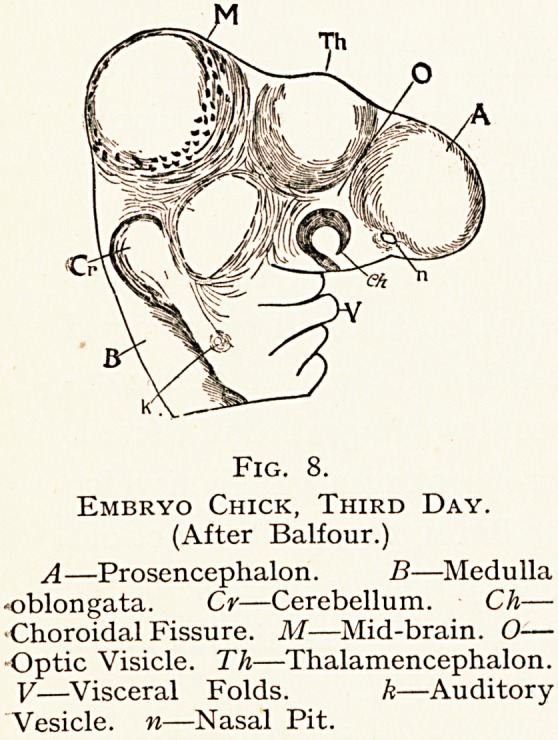


**Fig. 9. f9:**
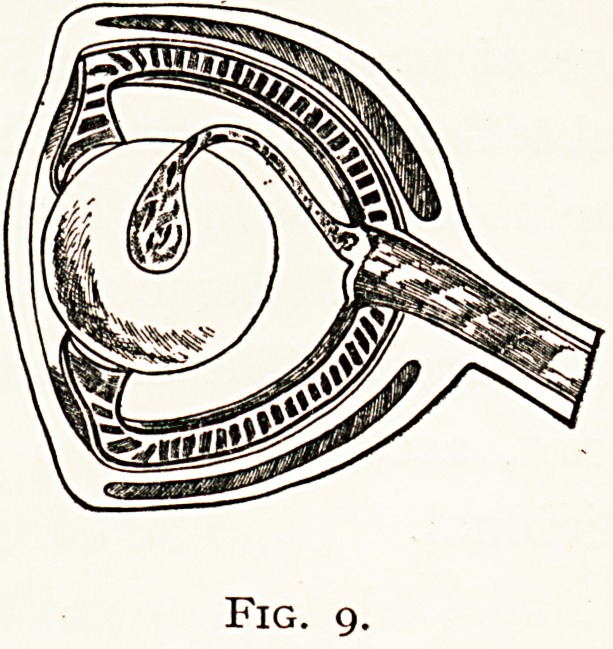


**Fig. 10. f10:**
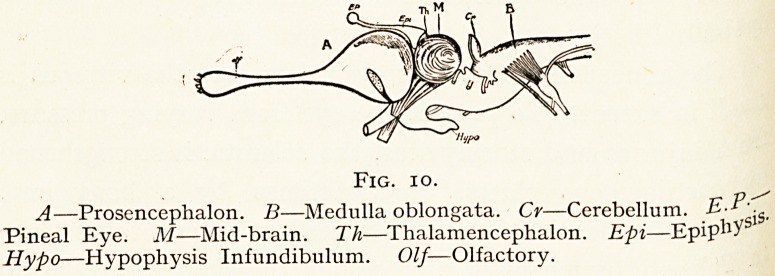


**Fig. 11. f11:**
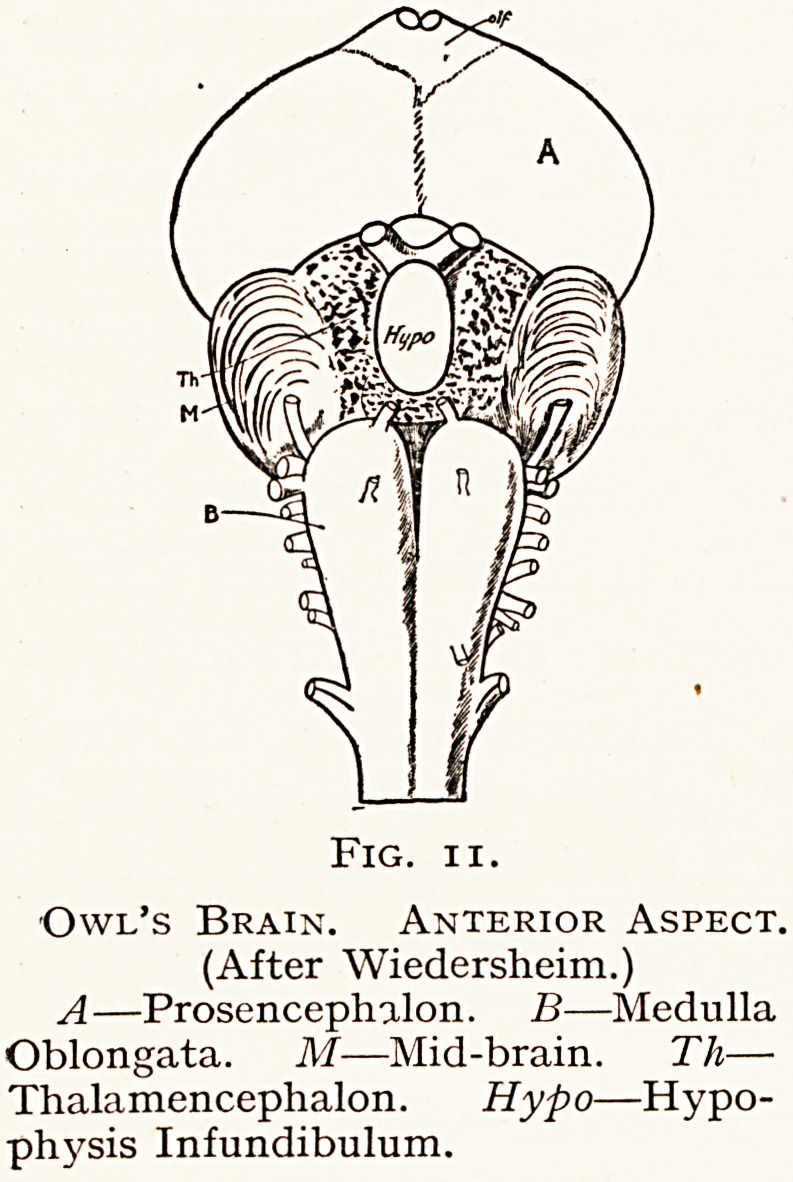


**Fig. 12. f12:**
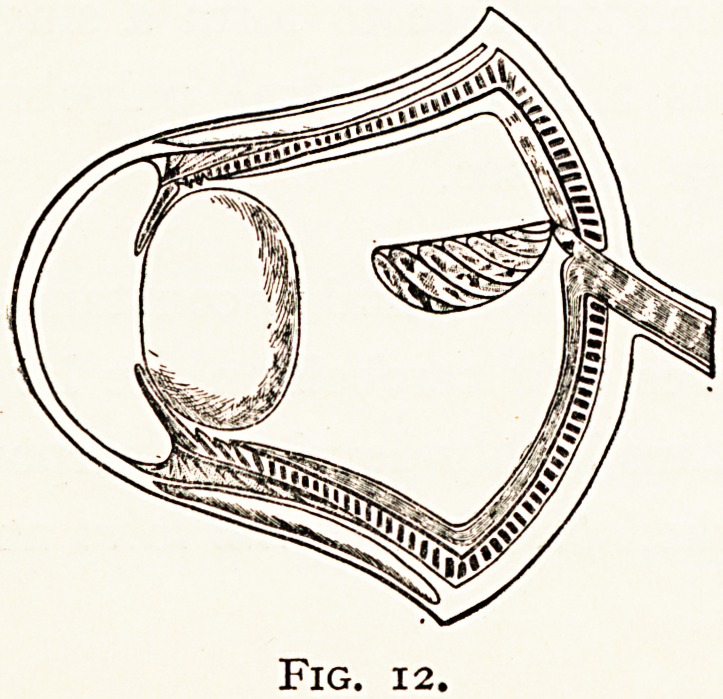


**Fig. 13. f13:**
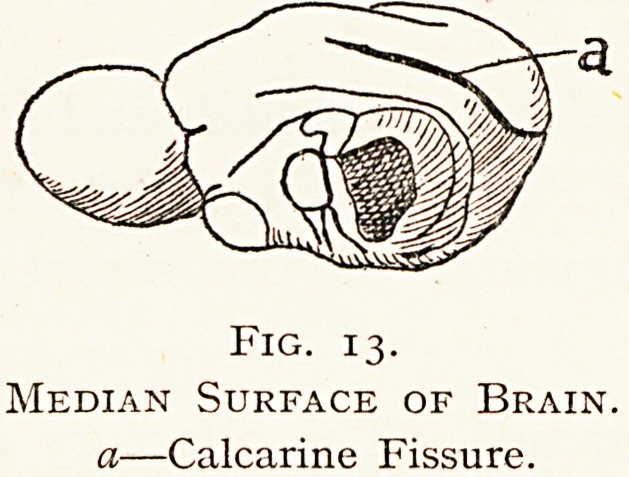


**Fig. 14. f14:**
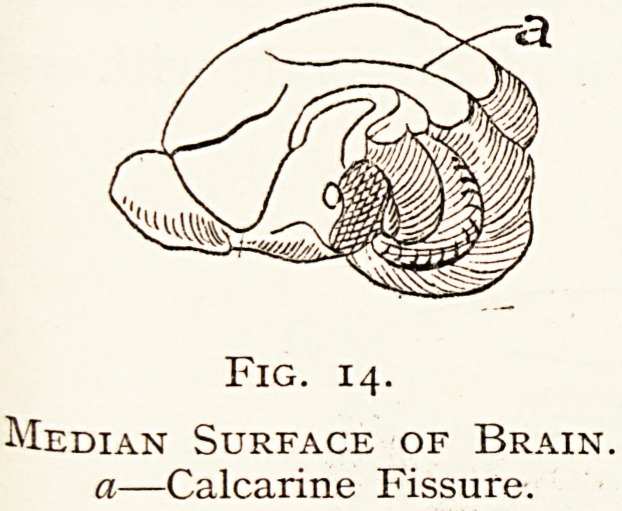


**Fig. 15. f15:**
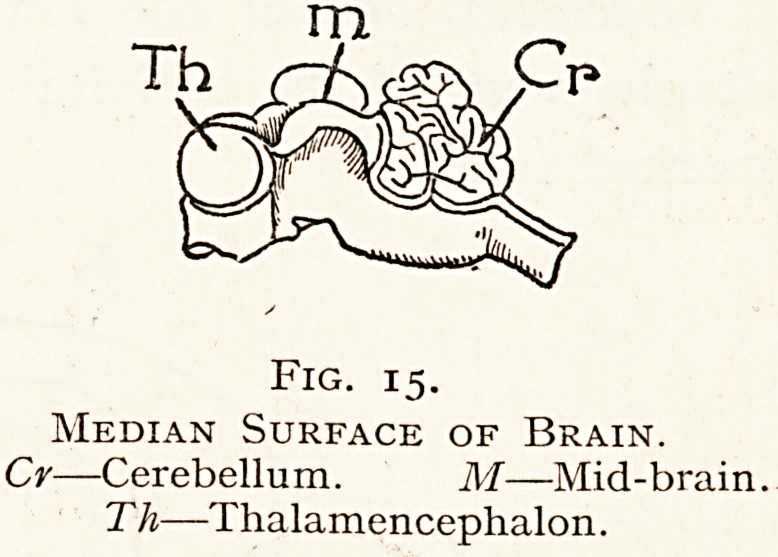


**Fig. 16. f16:**
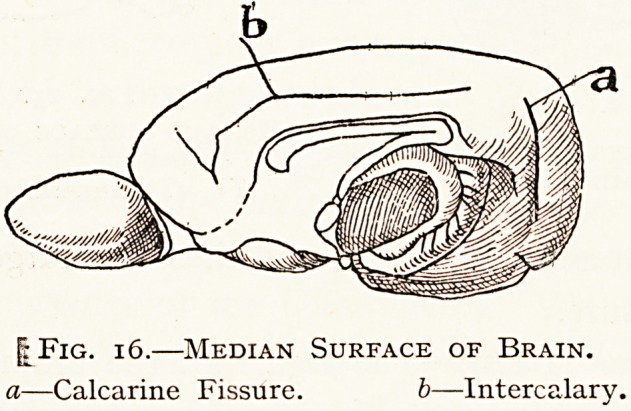


**Fig. 17. f17:**
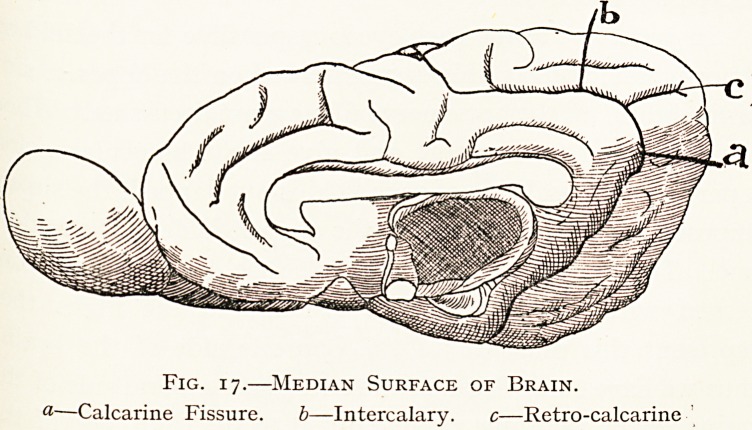


**Fig. 18. f18:**
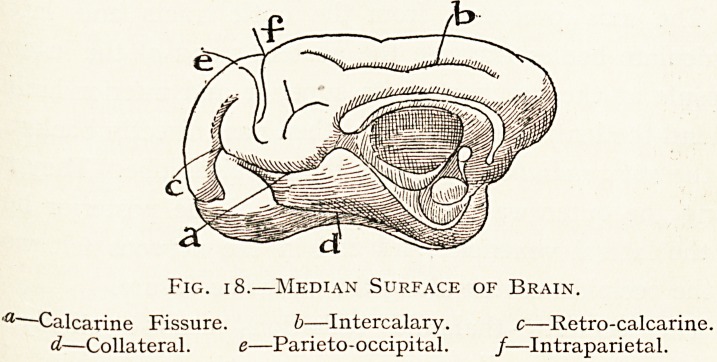


**Fig. 19. f19:**